# Correlation between “Snake-Eyes” Sign and Role of Surgery with a Focus on Postoperative Outcome: A Systematic Review

**DOI:** 10.3390/brainsci13020301

**Published:** 2023-02-10

**Authors:** Gianluca Scalia, Roberta Costanzo, Lara Brunasso, Giada Garufi, Lapo Bonosi, Giuseppe Ricciardo, Francesca Graziano, Giovanni Federico Nicoletti, Salvatore Massimiliano Cardali, Domenico Gerardo Iacopino, Rosario Maugeri, Giuseppe Emmanuele Umana

**Affiliations:** 1 Neurosurgery Unit, Head and Neck Surgery Department, Garibaldi Hospital, 95123 Catania, Italy; 2 Neurosurgical Clinic, AOUP “Paolo Giaccone”, Post Graduate Residency Program in Neurologic Surgery, Department of Biomedicine Neurosciences and Advanced Diagnostics, School of Medicine, University of Palermo, 90127 Palermo, Italy; 3 Department of Neurosurgery, Mayo Clinic, Rochester, MN 55905, USA; 4 Department of Neurosurgery, Azienda Ospedaliera Papardo, University of Messina, 98158 Messina, Italy; 5 Division of Neurosurgery, BIOMORF Department, University of Messina, 98125 Messina, Italy; 6 Cannizzaro Hospital, Trauma Center, Gamma Knife Center, 95125 Catania, Italy

**Keywords:** spine, cervical, myelopathy, cobra-eyes, surgery, outcome

## Abstract

(1) Background: The “snake-eyes” sign represents a unique finding characterized by bilateral hyperintense symmetric, circular, or ovoid foci on T2-weighted MRI sequences in the anterior horn cells of the spinal cord. There are conflicting opinions as some authors affirm that it does not affect the prognosis of cervical myelopathy while other papers emphasize the opposite, stating how the “snake-eyes” sign constitutes an irreversible lesion and a predictor of poor prognosis. This systematic review evaluates the correlation between the “snake-eyes” sign and the prognosis of cervical myelopathy after surgery including anterior and/or posterior approaches; (2) Methods: A systematic literature review was conducted following the PRISMA statement and a total of seven papers were included; (3) Results: A total of 419 patients were evaluated, with a mean age of 55.72 ± 14.38 years. After surgery, 26.01% of patients experienced a significant clinical improvement, while in 61.81%, there was no significant improvement. In particular, 144 of 196 patients (73.5%) treated through an anterior approach and 114 of 223 (51.1%) that underwent a posterior approach, did not present a significant improvement. Furthermore, in 12.17% of patients, the postoperative outcome was not reported, leading to a high risk of bias in the assessment of the prognostic significance of the “snake-eyes” appearance; (4) Conclusions: The “snake-eyes” sign is usually considered as an unfavorable predictive marker for myelopathic surgical patients, but the pathophysiology is still unclear, and the results have not yet reached unified levels of evidence.

## 1. Introduction

Degenerative cervical myelopathy (DCM) is a debilitating, progressive and chronic condition characterized by neurological impairment due to static and dynamic injury of the cervical spine [1]. It represents the most common cause of spinal cord dysfunction due to the progressively aging population and comprises several clinicopathological entities such as cervical spondylotic myelopathy (CSM), ossification of the posterior longitudinal ligament (OPLL), ossification of the ligamentum flavum (OLF), and degenerative disc disease (DDD) [2,3]. The pathophysiological mechanism lies in the progressive microvascular spinal cord compression, resulting in regional spinal cord blood flow alteration and reduction, with consequent ischemic phenomena. Furthermore, the breakdown of the blood–spinal cord barrier (BSCB) and the reactive inflammation cascade plays a pivotal role in determining an alteration of ion homeostasis mechanisms, thus resulting in axonal and glial apoptosis [4]. Clinical onset is often insidious, leading to a delay in the diagnosis with a significant impact on prognosis.

It has been shown that early diagnosis and surgical management might improve neurologic and overall outcomes and prevent further deterioration [5,6]. Since the pattern of symptoms is often subtle and non-specific, the final diagnosis of DCM requires imaging evidence of cervical spine compression related to one or more symptoms or signs caused by cervical myelopathy [7]. Magnetic resonance imaging (MRI) represents the gold standard for evaluating spinal cord compression and the most valuable MR sequences are T2-weighted images, which compare the signal ratio of the white matter to gray matter [8]. Several studies showed that signal hyperintensities in T2-weighted MR sequences were found in 41 to 97% of patients with DCM [9,10]. In addition, in recent years, new data analysis algorithms have been developed to detect presymptomatic spinal cord injury. For instance, it has been demonstrated using spinal cord shape analysis and multiparametric quantitative MRI to identify spinal cord damage before the onset of neurological symptoms and signs [11]. Advanced MRI techniques have increasingly demonstrated clinical utility. In particular, diffusion tensor imaging (DTI) parameters such as fractional anisotropy (FA) and apparent diffusion coefficient maps (ADC) are of great value, with a lower FA and higher apparent diffusion coefficient typically present in patients with myelopathy [12,13].

Early decompressive surgery, either with an anterior and/or posterior approach, is the gold standard treatment for DCM. The choice of surgical strategy is dictated by the underlying pathology and the surgeon’s expertise. In this scenario, the current challenge is to identify patients in the early stages of disease and, therefore, select the best candidates for surgical treatment, basing the surgical strategy on reliable clinical and radiological markers [14,15].

One of the most debated radiological signs is the so-called “snake-eyes” sign, also known as “owl-eyes” or “fried-eggs” sign. It represents a unique finding appearing as bilateral hyperintense symmetric, circular, or ovoid foci on T2-weighted MR axial sequences in the anterior horn cells of the spinal cord [16]. The pathogenetic theory underlying this sign is still unclear; however, the most accepted hypothesis seems to be based on damage or obstruction of the anterior spinal artery, which provides the main blood supply to the anterior two-thirds of the spinal cord, as reported by many case reports [17,18,19,20]. From an anatomopathological point of view, the main modifications are cystic necrosis at the junction of the central grey matter and the posterior ventrolateral column combined with cell loss in the anterior horn [21]. The precise prognostic significance of the “snake-eyes” sign is still unclear. Some authors postulated that it does not affect the prognosis of myelopathy [22,23], while other studies emphasized the opposite, stating how the “snake-eyes” sign constitutes an irreversible lesion and a predictor of poor prognosis [24,25].

The aim of this systematic review is to evaluate the postoperative outcome and the eventual correlation between the “snake-eyes” sign and the prognosis of cervical myelopathy after surgical procedure (i.e., anterior cervical discectomy and fusion (ACDF) and/or posterior approaches, comprising laminectomy with or without fusion and laminoplasty).

## 2. Materials and Methods

**Figure 1:**
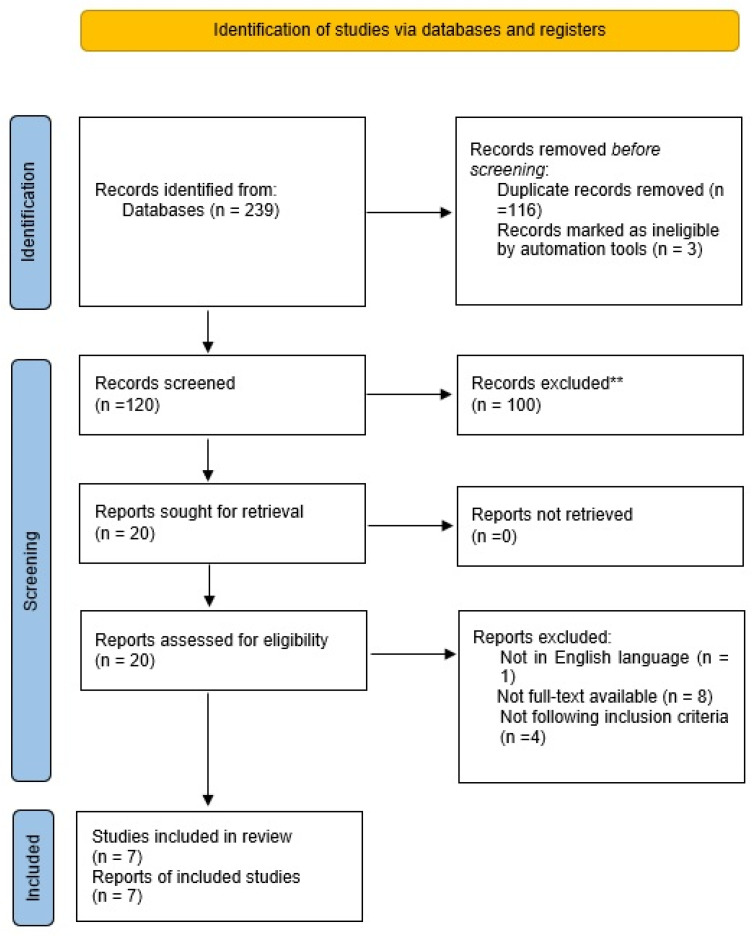
PRISMA flow diagram reports the decision algorithm for the selection of the studies of the systematic review.

A systematic literature review was conducted following the PRISMA (Preferred Reporting Items for Systematic Reviews and Meta-Analyses) statement, with no limits in terms of publication date. (Figure 1) Recorded studies were exported to Mendeley software. Only articles in English language were included. All the duplicates were removed, and a manual search was also performed to identify eventual additional studies of the reference sections. Two reviewers (G.S. and R.C.) independently screened the titles, abstracts, and full manuscripts, then the results were combined and analyzed. The following Mesh and free text terms were identified: “myelopathy AND owl-eyes” (10 results), “myelopathy” AND “snake-eyes appearance” (15 results), “myelopathy” AND “snake-eyes sign” (3 results), “myelopathy” AND “owl eye signs” (5 results), “myelopathy” AND “owl eyes appearance” (3 results), “cervical surgery” AND “snake eyes” (11 results), “cervical surgery” AND “owl eyes” (4 results), “cervical surgery” AND “snake-eyes appearance” (8 results), “cervical surgery” AND “owl eyes sign” (1 results), “cervical surgery” AND “snake eyes sign” (4 results), “spondylosis” AND “snake eyes” (4 results), “spondylosis” AND “fried-eggs signs” (1 result), “spondylosis” AND “snake eyes sign” (1 result), “spondylosis” AND “snake eyes appearance” (4 results), “prognosis” AND “snake eyes sign” (4 results), “prognosis” AND “owl eyes sign” (2 results), “prognosis” AND “snake eyes appearance” (7 results), “prognosis” AND “owl eyes appearance” (6 results), “MRI” AND “snake eyes” (47 results), “MRI” AND “snake eyes sign” (9 results), “MRI” AND “owl eyes sign” (14 results), “MRI” AND “owl eyes appearance” (5 results), “MRI” AND “owl eyes” (27 results), “MRI” AND “snake eyes appearance” (26 results).

### 2.1. Eligibility Criteria

The articles were selected according to the following inclusion criteria:

- Full article in English
- Clinical studies (case reports, case series, retrospective studies)
- Studies with description of “snake-eye” appearance myelopathy related to degenerative diseases (as cervical stenosis and/or spondylosis)
- Studies focusing on patients with “snake-eye” appearance surgically treated.

### 2.2. Exclusion Criteria

- Articles not in English
- Literature reviews, and meta-analysis
- Patients with “snake-eyes” sign myelopathy due to vascular injuries (i.e., Hirayama disease)
- Studies not reporting a surgical treatment.

### 2.3. Data Extraction

The available data included authors, year, study design, number of patients, sex, age, myelopathy location, pre- and postoperative symptoms, surgical approach, postoperative MRI (if available), follow-up and postoperative course. Patients’ demographics are shown in Table 1.

## 3. Results

A total of 239 published studies were identified through PubMed, Google Scholar, Scopus databases and additional reference list searches. After removing duplicates, the papers screened were n = 120. Based on the titles and abstracts, we then excluded 100 articles. Eight articles were not retrieved due to the unavailability of the full text, one article was excluded because not in English language. We excluded the other four papers due to incompatibility with our eligibility criteria. Finally, a total of seven papers were included in this systematic review.

Patients’ characteristics, demographics, and treatment information were not uniformly or consistently reported across the included studies. Papers were selected in this review with no limit of time, including studies from 1985 to 2021. A total of 419 patients were evaluated, with a mean age of 55.72 ± 14.38 years. Of the patients, 297 patients were males (70.88%) and 122 were females (29.11%) with a female/male ratio of 2.43/1. One hundred and ninety-six patients were surgically treated using an anterior approach (46.77%), while 223 patients (53.22%) underwent a posterior approach. The cervical metamer mostly involved was C5–C6. A proportion of 64.2% of patients included in this review reported motor disturbances as preoperative clinical symptoms, ranging from upper limb or leg weakness, spasticity to complete tetra or paraparesis associated with 29.35% of cases, to sensory disturbances, involving especially the fingers and hands.

After surgery, 26.01% of patients experienced a significant clinical improvement, while in 61.81% there was no significant improvement. In particular, 144 of 196 patients (73.5%) that were treated through an anterior approach and 114 of 223 (51.1%) that underwent a posterior approach did not present a significant improvement. Furthermore, in 12.17% of patients, the postoperative outcome was not reported, leading to a high risk of bias in the assessment of the “snake-eyes” sign’s prognostic significance. In only three papers was a detailed 6-month follow-up reported, with a poor outcome, not improved after ACDF (JOA 12.9 ± 1.3) and two patients improved in upper limb strength after laminectomy and fusion.

In three out of seven papers (249 patients, 59.4%), postoperative imaging (spine MRI) was reported, and the snake-eyes sign continued to be detected after surgery.

## 4. Discussion

### 4.1. Neuroradiological Features

The typical “snake-eyes” sign is a unique neuroimaging finding characterized by two symmetrical high-signal rounds on the spinal parenchyma resembling the face and eyes of a snake on T2-weighted MR images, located on the central gray matter near the ventrolateral posterior columns [29].

Despite the concept of the snake-eyes appearance (SEA) introduced by Jinkins and Al-Mefty in 1986 [30], similar images described as “high-density areas resembled fried eggs” in the gray matter were first documented by Iwasaki et al., in CT scans and myelographies several years before [26]. The authors also showed the usefulness of CT scans in patients with CSM by demonstrating the degree and direction of spinal cord compression by the osteophytes and discs, the severity of deformation or the spinal cord atrophy, and the eventual pincer effect on the dynamic movements of the spine. The presence of intramedullary high-signal intensity includes a wide spectrum of pathological changes derived from the compression of the spinal cord, with both reversible (edema) or irreversible changes (gliosis, myelomalacia, cystic necrosis, syrinx formation), and is frequently seen in patients with cervical compressive myelopathy, such as degenerative cervical spondylosis or OPLL [22,25,31].

### 4.2. Pathophysiology

The snake-eyes sign is associated with necrosis in the gray matter of the spinal cord due to chronic spinal cord compression [21,22,25,28], in contrast to the reversible nature that some authors claimed in the past [32]. This sign is also described in association with different neurological conditions such as anterior spinal artery ischemia, monomelic amyotrophy of the upper limb, amyotrophic lateral sclerosis, spinal muscular atrophy, and Hirayama disease [33]. The clinical role and pathophysiology are still not well elucidated [16]. Chronic mechanical compression and vascular impairment are considered the main promoters for its development, for example, during the late stage of Hirayama disease, as derived from local ischemia in the anterior horn triggered by arterial compression during anterior–posterior shifting of the cervical spine in the neck flexion and extension movements [33].

### 4.3. Diagnosis

Spine MRI represents the gold standard for documenting the spinal cord morphology and its pathologic alterations. Signal MRI changes in the spinal cord are considered a useful index for indirectly evaluating the severity of the neurological status in patients with DCM, and for predicting outcomes, especially MRI T2-weighted images with higher-signal intensity change suggesting more severe myelopathy and a potentially poorer prognosis [27,28]. Preoperative low signal intensity changes on MRI T1-weighted images have also been associated with postoperative outcomes in the literature as a sign of advanced disease [16,28,29]; some authors describe that signal intensity changes in T1-weighted images are usually related with an increase in T2-weighted images, and, thus, T2 hyperintensity alone could not be a solely predictor for poor postoperative outcome, encouraging the evaluation of both signal alterations on T1-weighted images and T2-weighted images [34]. However, there is still no definitive evidence associated with predicting outcomes from the preoperative evaluation of the severity of DCM and MRI findings [15,16,21,24,28,35] in both surgical and non-surgical patients.

Recently, Funaba et al. [31] aimed to elucidate the relationships between signal intensity changes on MRI and cervical spine alignment, balance, spinal cord compression, and dynamic changes in the spinal cord. They documented that severe and poorly compensated spinal cord compression, particularly during neck flexion, was more likely to cause MRI signal intensity changes, even in the absence of cervical spondylosis. Moreover, they concluded that patients with the snake-eyes sign resulted in significantly inferior postoperative upper extremity function, probably due to irreversible gray matter damage [21,27], as well as those cases with multilevel high-signal intensity changes on T2-weighted images showing inferior postoperative recovery of lower limb function and lower limb spasticity [28,36], probably due to the irreversible damage caused by severe degeneration [37]. These results were in line with previous studies reporting higher-signal intensity being more frequently associated with inadequate postoperative high-signal intensity regression [15,28]. Mohanty et al., reported the combination of larger cervical curvature, (lordosis and kyphosis) with severe myelopathy, and greater high-signal intensity changes on T2-weighted images [38].

### 4.4. Surgical Treatment and Postoperative Outcome

As is already widely known, the anterior approach through the ACDF and the posterior approach of performing a laminoplasty or a laminectomy plus/minus fusion for CSM have been extensively studied and discussed in the literature. Furthermore, the choice of anterior or posterior approach in multilevel CSM remains controversial and debated [1,4].

Shi et al., compared the neurological and radiographic outcomes of multilevel (four) CSM treated by performing laminoplasty and ACDF, without any reference to the snake-eyes sign on the preoperative spine MRI. They stated that postoperative neck disability index (NDI) scores and neck pain visual analogue scale (VAS) scores in the ACDF group were significantly lower than those in the posterior approach group at 3-year follow-up [39].

Evaniew et al., in an observational study, found that the most significant neurological improvement after surgery for CSM occurred by 3 months, while the most significant improvements in pain and health-related QoL occurred within 3 months or 1 year after surgery [40].

Mizuno et al., showed that the preoperative upper-extremity motor dysfunction grade was significantly higher in patients with multilevel intramedullary high-signal intensities than in patients with single-level intramedullary high-signal intensity or in the absence of any hyperintensities [21]. The incidence of significant upper-limb motor weakness was 67% for multilevel SEA; 20% for single-level SEA; 32% with multilevel non-SEA; 11% with single-level non-SEA. These results suggest that multilevel SEA leads to the development of upper-extremity weakness. Postoperative results showed that SEA continued to be present postoperatively in all cases, supporting the irreversible nature of this phenomenon.

There was a significant difference in the reported recovery rate between SEA and non-SEA groups and between SEA and the control groups, although there was no positive difference in the recovery rate between the latter groups. Similar results were reported by Fontanella et al., in their case series: after surgery (anterior decompression plus laminectomy and fusion) the SEA was still detected in the postoperative MRI. Conversely, this group showed a slight improvement in upper limb strength in two out of three patients, despite the postoperative radiological evidence of SEA [16].

Iwamae et al., retrospectively reviewed 103 patients who underwent cervical laminoplasty in terms of residual upper extremity (UE) numbness: they found that patients with residual UE numbness showed minimal improvements in postoperative neck pain [27]; risk factors were also considered: in particular, female sex and preoperative severe UE pain were negative predictive factors for residual UE numbness. In any case, no significant correlation was found with preoperative SEA between residual UE numbness groups. These findings suggest that clinical outcome after surgery is affected by several independent variables, such as age, sex, and body mass index (BMI); the impact of preoperative neuroradiological findings such as the cobra-eyes sign after surgery on clinical outcome is still broadly debated.

Several studies investigated the correlation between the preoperative evidence of modified signal intensities on imaging including the snake-eyes sign, neurological preoperative presentation and surgery performed with controversial results. Zhang et al. [35] identified cervical instability and anterior compression as factors exacerbating postoperative high-signal intensities in patients with degenerative cervical myelopathy who underwent laminoplasty without instrumented fusion and suggested that combined fixation or anterior decompression and fusion need to be considered for these patients. Funaba et al., analyzed patients who underwent instrumented fusion, concluding that the preoperative SEA and multilevel intramedullary high-signal intensities on T2-weighted images may have been useful for predicting postoperative outcomes [31].

Conversely, Choi et al., analyzed 34 patients with OPLL diagnosis with SEA on their preoperative spine MRI and documented a trend toward good prognosis rather than poor prognosis with no clear statistical significance [22]. They concluded that SEA did not significantly affect the prognosis of patients who underwent anterior cervical corpectomy and fusion (ACCF) for OPLL treatment.

### 4.5. Study Limitations

There are few studies corresponding to the inclusion criteria, so this does not allow a detailed statistical analysis, but only a qualitative analysis of the available data. Furthermore, it is not possible to compare the type of surgical approach (anterior and/or posterior) with the postoperative outcome in patients with the “snake-eyes” sign due to the limited results obtained and because no detailed analysis was performed in any of the selected studies.

## 5. Conclusions

As evidenced by the literature review, the snake-eye appearance is usually considered as an unfavorable predictive marker for myelopathic surgical patients, but the pathophysiology is still unclear, and the results have not reached unified levels of evidence. Thus, further, larger volume of date results may help in providing adequate information for patients with cervical compressive myelopathy and help surgeons in predicting poor surgical outcomes preoperatively.

## Figures and Tables

**Table 1:** Systematic review of authors, year, study design, number of patients, sex, age, myelopathy location, pre- and postoperative symptoms, surgical approach, postoperative MRI, follow-up, and postoperative course of patients affected by “snake-eyes” sign myelopathy reported in the literature to date.

Authors	Number of Patients	Sex	Age	Myelopathy Location	Neurological Status before Surgery	Surgical Approach	Neurological Status after Surgery	Follow-Up	Postoperative MRI
Iwasaki et al., 1985 [26]	1	M	55	C4–C5	Numbness of left fingers. Spasticity of both legs	Anterior approach	Improved	NOT REPORTED	NOT REPORTED
	1	M	65	C4–C5, C5–C6	tetraparesis (rt > lt). Sensory disturbance below T4 on lt	Anterior approach	Improved	NOT REPORTED	NOT REPORTED
	1	M	52	C4–C5, C5–C6	numbness of right extremities. Left hemiparesis and sensory disturbance below T4 on right	Laminectomy and fusion	Moderate improvement	NOT REPORTED	NOT REPORTED
	1	M	51	C4–C5, C5–C6, C6–C7	Leg spasticity and motor weakness of right hand	Laminectomy	NOT REPORTED	NOT REPORTED	NOT REPORTED
	1	F	68	C3–C4, C4–C5, C5–C6	urinary disturbance, Tetraparesis, muscle atrophy of arms	Laminectomy	NOT REPORTED	NOT REPORTED	NOT REPORTED
	1	F	61	C3–C4, C5–C6, C6–C7	numbness of fingers. Rt hemiparesis, hyperreflexia	Anterior approach	NOT REPORTED	NOT REPORTED	NOT REPORTED
	1	F	67	C3–C4, C4–C5, C5–C6	numbness of fingers. Motor weakness of rt hand, generalized hyperreflexia	Laminectomy and fusion	NOT REPORTED	NOT REPORTED	NOT REPORTED
Mizuno et al., 2003 [21]	144	110 M, 34 F	56.5	level NS	Upper limb weakness (JOA 10.8 ± 1.7)	Anterior approach and fusion	Not improved after surgery	Poor outcome, not improved after surgery (JOA 12.9 ± 1.3)	Snake-eye appearance continued to be present
Mizuno et al., 2005 [25]	1	M	73	C4–C6	moderate weakness of the deltoid, biceps, triceps and grip strength with muscle atrophy, hyperreflexia in the knee and ankle, jerks, hypesthesia in C5–T1 dermatome, severe gait difficulty	Anterior approach and fusion	Not improved after surgery	Sudden death of acute myocardial infarction after 3 months	NOT REPORTED
Choi et al., 2005 [22]	47	36 M, 11 F	54.7	level NS	NOT REPORTED	Anterior approach and fusion	NOT REPORTED	Good prognosis	NOT REPORTED
Iwamae et al., 2020 [27]	103	65 M, 38 F	65.5	level NS	NOT REPORTED	Laminectomy	Improvement	NOT REPORTED	Snake-eye appearance continued to be present
Fontanella et al., 2020 [16]	1	M	21	C5–C6	Upper extremities hypoesthesia and pain, especially in the right arm and mild weakness of wrist extensor muscles, again worse on the right side, with sporadic dropping of objects	Anterior approach and fusion	Upper extremities motor and sensory Improvement	NOT REPORTED	NOT REPORTED
	1	M	44	C5–C6	Upper limbs diparesis and hypoesthesia	Laminectomy and fusion	Improvement	6 months improvement of strength in his arms	Snake-eye appearance continued to be present
	1	F	56	C5–C6	Pain in both arms and weakness of distal right arm movements	Laminectomy and fusion	Pain reduced	6 months complete recovery of strength in her right hand	Snake-eye appearance continued to be present
Funaba et al., 2021 [28]	114	79 M, 35 F	67.9	level NS	Clumsy hands, numbness, gait disturbance, hyperreflexia, and pathological reflexes	Laminectomy and fusion	Not improved after surgery	NOT REPORTED	NOT REPORTED

## Data Availability

Not applicable.
